# Concurrent MLL-AF4^+^ infant ALL in monozygotic twins: a case report

**DOI:** 10.3389/fped.2026.1777726

**Published:** 2026-03-25

**Authors:** Chenghui Li, Xin Tong, Jinfeng Yang, Hui Gao, Zhong Li

**Affiliations:** 1 Dalian Women and Children’s Medical Group, Hematology-Oncology Department, Dalian, Liaoning, China; 2 Dalian Women and Children’s Medical Group, Department of Pharmacy, Dalian, Liaoning, China

**Keywords:** acute lymphoblastic leukaemia, CAR-T, infants, MLL-AF4, monozygotic twins

## Abstract

Acute lymphoblastic leukemia (ALL) is the most common malignancy in childhood, but it is relatively rare in infants. However, infant ALL exhibits distinct biological characteristics and an aggressive course, with a high proportion of cases involving MLL gene rearrangements (particularly the MLL-AF4 fusion gene). Leukemia driven by MLL-AF4 is extremely rare across all populations but represents a critical and poor-prognosis subtype within infant ALL.ALL driven by the MLL-AF4 fusion is exceptionally rare and particularly fulminant. We report monochorionic monozygotic twins who presented simultaneously with MLL-AF4 positive ALL. Both neonates exhibited fever, pallor, hepatosplenomegaly and extreme leucocytosis. After achieving complete remission with the St.Jude Total Therapy XV (TOTXV) protocol, the twins experienced molecular relapse and were successfully rescued with sequential CD19- and CD22-directed CAR-T-cell therapy. Owing to parental preference and financial constraints, neither patient proceeded to allogeneic haematopoietic stem-cell transplantation; instead, they received consolidative chemotherapy. At 8 years of age, both children remain in sustained molecular remission and enjoy age-appropriate development. This concordant twin pair illustrates the natural history of MLL-AF4-positive infant ALL, supports the curative potential of CAR-T-cell therapy in this ultra-high-risk population, and emphasises the importance of prolonged molecular surveillance.

## Highlights

- We report a rare case of synchronous MLL-AF4 infant acute lymphoblastic leukaemia occurring in monozygotic twins.
- This study establishes the long-term efficacy of CAR-T cell therapy in high-risk infants with MLL-AF4 acute lymphoblastic leukaemia.
- Underscores the central value of prolonged, intensive surveillance and serial MRD monitoring in the management of these patients.

## Introduction

Infant leukaemia is biologically distinct from all other leukaemia subtypes and constitutes only 2.5%–5% of childhood acute lymphoblastic leukaemia (ALL) (1). Population-based SEER*Explorer data indicate that the incidence of acute lymphoblastic leukemia in infancy is 1.8 cases per 100,000 people (2). The 5-year event-free survival remains 45%, significantly inferior to that of older children with ALL (3). Chromosomal translocations that generate fusion genes are central to the pathogenesis of ALL, dictating disease classification, risk assignment and therapeutic strategy. In infant ALL the MLL-AF4 fusion—created by t(4;11) (q21;q23)—is the most common driver lesion. This chimaeric transcript is virtually restricted to leukaemias arising in the first year of life and operates as the principal oncogenic initiator (4). Concordant infant acute lymphoblastic leukaemia (ALL) in monozygotic twins is exceedingly rare, with fewer than a dozen pairs documented worldwide. Such cases provide a unique window into the relative contributions of constitutional genetics and perinatal environmental exposures to leukaemogenesis. We describe a 6-month-old monochorionic twin who developed MLL-AF4-positive pro-B-ALL and attained sustained molecular remission after intensive chemotherapy, sequential CD19- and CD22-directed CAR-T-cell therapy, and maintenance chemotherapy without allogeneic haematopoietic stem-cell transplantation. This observation expands the limited evidence base for managing MLL-rearranged infant ALL and may inform risk-adapted, transplant-sparing strategies for similarly affected children.

## Case presentations

**Figure 1:**
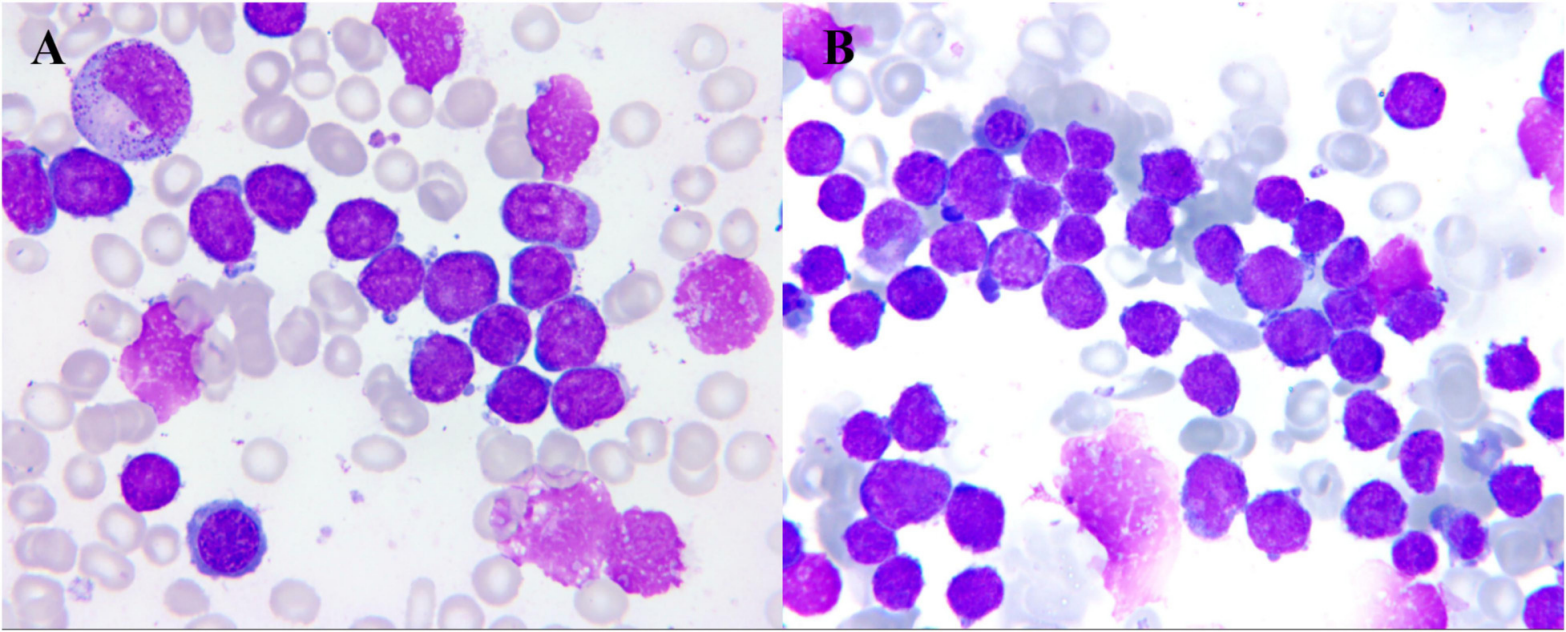
Representative bone-marrow smear **(A)** the second-born twin sister twin A: myeloproliferative disorder, stage II, characterized primarily by abnormal proliferation of the lymphoid system. Abnormal proliferation of the lymphatic system, with primary lymph nodes accounting for 85.5%. Observed cells exhibit variable cell body sizes, fine nuclear chromatin, visible nucleoli, and scant cytoplasmic blue staining. No megakaryocytes were observed throughout the entire slide. **(B)** The first-born twin sister Twin B: Myeloproliferative disorder, stage II, characterized primarily by abnormal proliferation of the lymphoid system. Abnormal proliferation of the lymphatic system, with primary lymph nodes accounting for 88.5%. The cell bodies are regular in shape, with fine nuclear chromatin and visible nucleoli. The cytoplasmic blue staining is sparse. Two granular megakaryocytes were observed throughout the entire slide.

A 6-month-old female infant (the second-born twin sister Twin A) was admitted after 1 day of fever and incidental laboratory abnormalities. Abdominal examination revealed mild distension; the liver was firm and palpable 2 cm below the right costal margin, and the spleen tip was palpable 4 cm below the left costal margin. At admission, the laboratory findings were white blood cells(WBC)level of 54.6 × 10⁹/L, lymphocyte percentage (LYM) level of 57.3%, platelets (PLT) level of 57 ×  10⁹/L, hemoglobin(Hb) level of 69 g/L. Bone-marrow aspirate showed marked lymphoblast hyperplasia; primitive lymphoid cells accounted for 85.5% of nucleated elements, consistent with grade II marrow involvement (Figure 1A) Twelve hours after the patient's admission, her monozygotic twin(the first-born twin sister Twin B) was admitted with a 15-day history of pallor and incidental cytopenias. Abdominal examination revealed mild distension; the liver was firm and palpable 3 cm below the right costal margin, and the spleen tip was firm and palpable 4 cm below the left costal margin. Laboratory data was WBC level of 126.9 × 10⁹/L,LYM level of 84.5%, Hb level of 73 g/L, PLT level of 53 × 10⁹/L. Bone-marrow aspirate showed grade II hypercellularity with diffuse lymphoblast expansion; primitive lymphoid cells constituted 88.5% of nucleated elements (Figure 1B).

**Figure 2:**
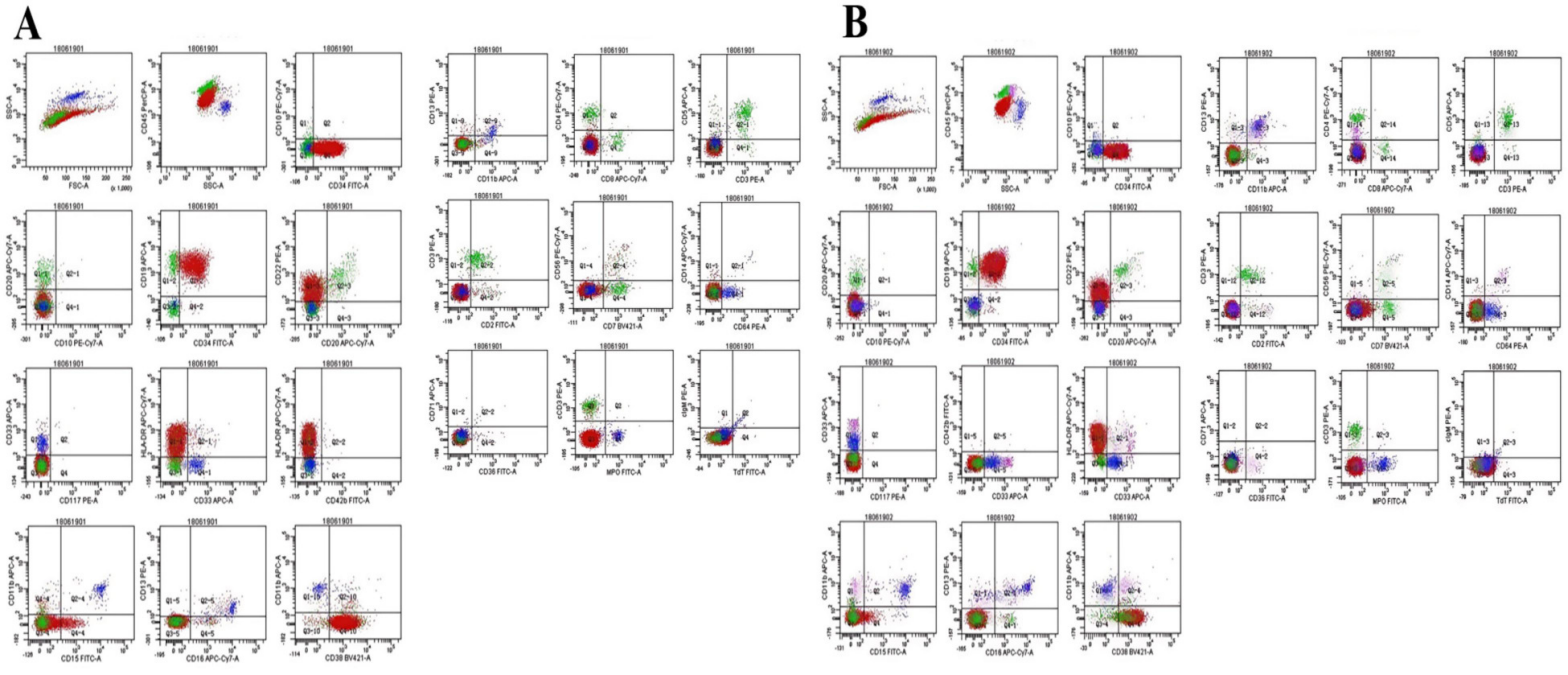
Bone marrow flow cytogram **(A)** the second-born twin sister Twin A **(B)** the first-born twin sister Twin B.

**Figure 3:**
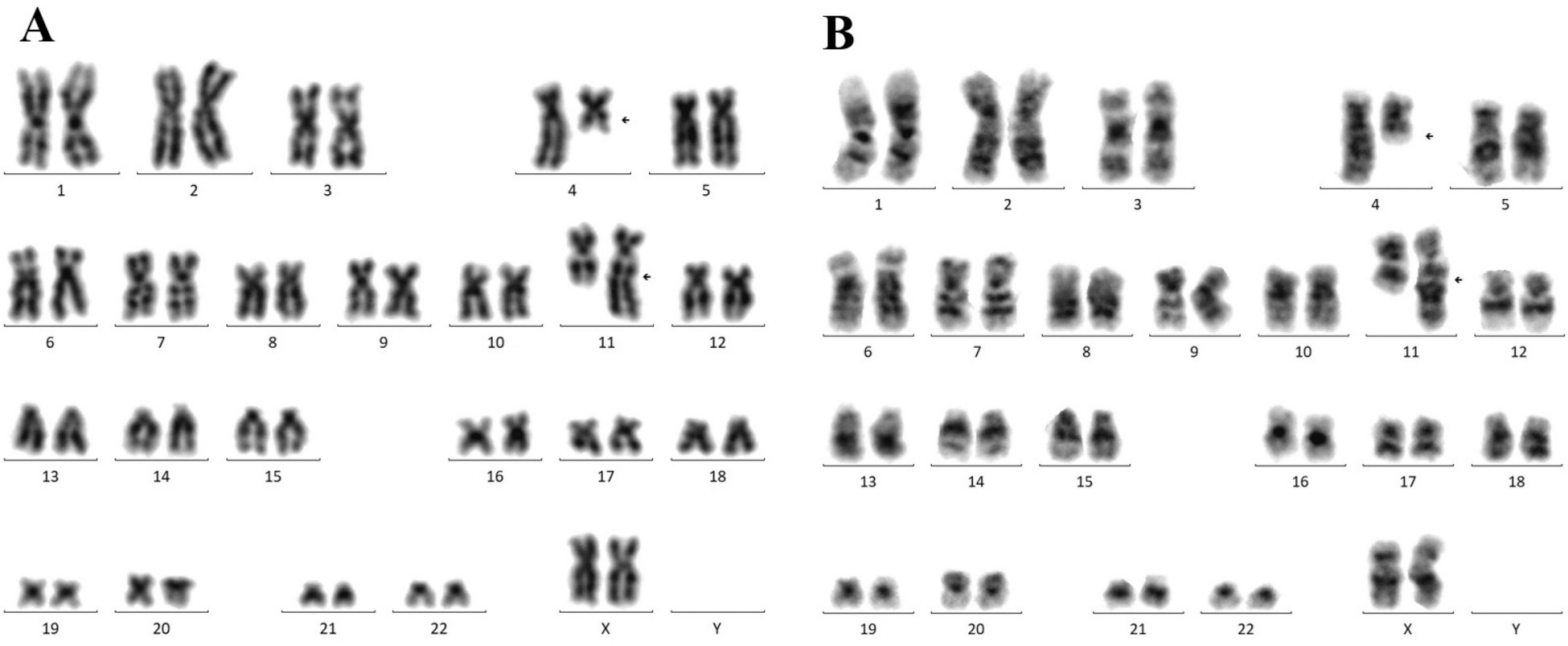
Chromosome testing **(A)** the second-born twin sister Twin A **(B)** the first-born twin sister Twin B.

Immunophenotyping (twin A): A CD45/SSC gate identified a blast population with dim CD45 and low SSC that comprised 87.2% of nucleated cells. The cells expressed HLA-DR, CD19, CD22, CD34 and CD38, lacked CD10 and cytoplasmic IgM, and were consistent with pro-B acute lymphoblastic leukaemia (Figure 2A). Immunophenotyping (twin B): Gating on CD45/SSC revealed a blast population with dim CD45 and low SSC that comprised 81.8% of nucleated cells. The cells expressed HLA-DR,

CD19, CD22, CD34 and CD38, lacked CD10 and cytoplasmic IgM, and were consistent with pro-B acute lymphoblastic leukaemia (Figure 2B). Screening for leukaemia-associated fusion genes confirmed the presence of MLL::AF4 in both twins (Figure 3).

### Treatment experience

Twin A was started on the VDLD + CAM induction regimen immediately after diagnosis (Table 1).

**Table 1.** Treatment experiences of Twins A and B.

Treatment Stage	Twin A	Twin B
Therapeutic approach	VDLD+CAM	
Day 19 bone marrow MRD follow-up	0.03%	0.68%
Day 26 bone marrow MRD follow-up	MDR negative	0.46%
Day 46 bone marrow MRD follow-up	MRD negative, MLL-AF4 gene negative	0.05%, MLL-AF4 gene negative
Follow-up after 4 cycles of HD-MTX+6-MP extramedullary prophylaxis chemotherapy regimen	MDR:2.23%, MLL-AF4 gene negative	MDR:0.1%, MLL-AF4 gene negative
Follow-up examination after chemotherapy with the (Dex+Ara-c+VP16+ASP) remission regimen	MDR:4.04%, MLL-AF4 gene positive	MDR:0.005%, MLL-AF4 gene negative
Follow-up treatment	After receiving CD19-CAR-T and CD22-CAR-T therapy at an external hospital, the patient returned to our institution for continued consolidation therapy (DEX + DOX + VCR + 6MP + ASP + HD-Ara-C).	Intensive salvage chemotherapy was continued for 20 weeks (DEX + DOX + VCR + 6MP + ASP+HD-Ara-c).
Maintenance treatment	CTX+Ara-c+MTX+6MP	
Results	Therapy was discontinued after 146 weeks. The patient is now 8 years old and in excellent general condition; the most recent evaluation on 10 July 2024 confirmed continued molecular remission.	Therapy was discontinued after 146 weeks. Follow-up examination 1.5 years post-discontinuation showed MRD 0.11% and MLL/AF4 gene expression 0.67%. Treated with Obinutuzumab. Continued with relapse regimen chemotherapy (Bortezomib + CTX + Aar-c + 6MP). After receiving CD19-CART therapy at another institution, achieved MRD and gene negativity, followed by sequential CD22-CART therapy. The most recent evaluation on 8 November 2025 confirmed continued molecular remission.

On day 19, minimal residual disease (MRD) in bone marrow was detected at 0.03%; it was subsequently cleared. By day 46, both MRD and MLL-AF4 fusion transcripts were undetectable.

After four cycles of high-dose methotrexate plus 6-mercaptopurine for extramedullary prophylaxis, MRD rebounded to 2.23%, whereas MLL-AF4 remained negative. Re-induction with dexamethasone, cytarabine, etoposide, and asparaginase was administered; MRD and MLL-AF4 both converted to positive. Six months after diagnosis, the patient received CD19-targeted CAR-T therapy followed by CD22-targeted CAR-T therapy, ultimately achieving complete molecular remission. Allogeneic transplantation was not pursued because of financial limitations. Consolidation and maintenance courses were completed in hospital, for a total treatment duration of 146 weeks. Treatment was discontinued, and the patient has remained molecularly negative and in good general condition through the last follow-up (10 July 2024).

**Figure 4:**
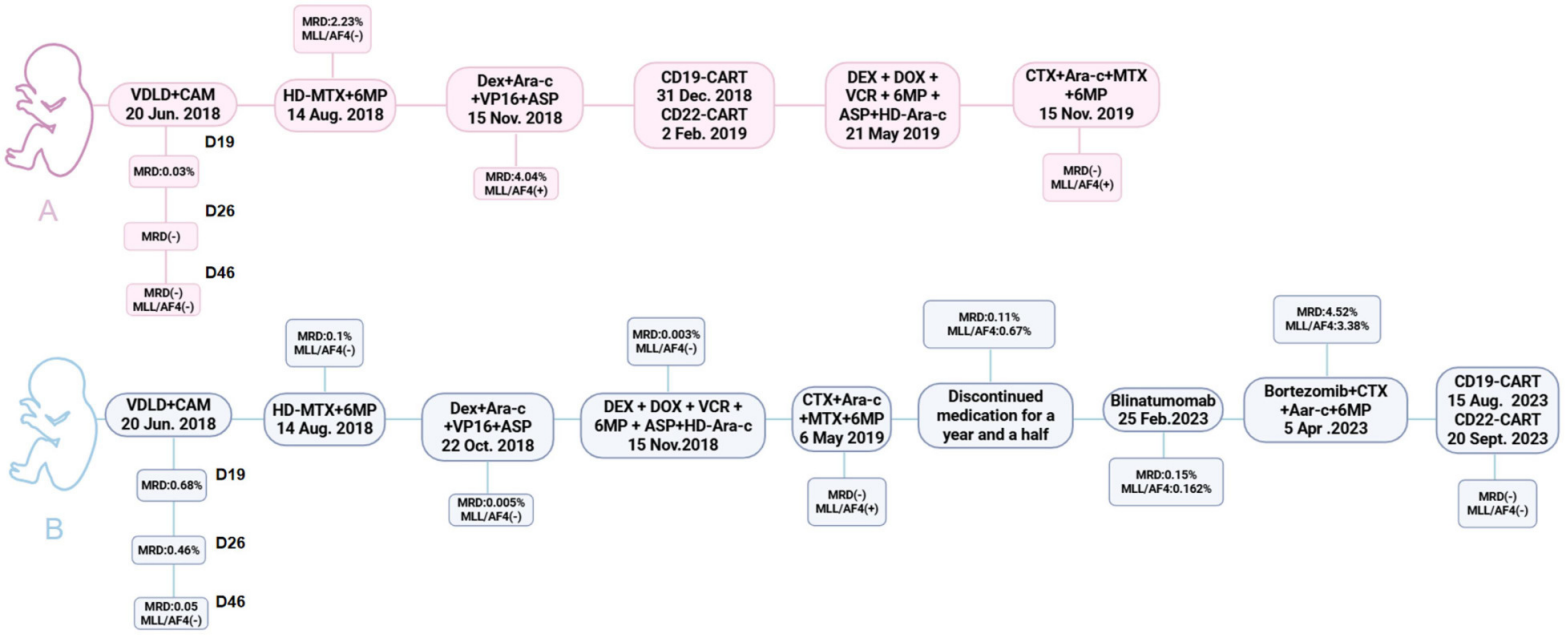
The treatment process for both individuals.

After VDLD + CAM induction, MRD in Twin B was sequentially reduced to 0.05% by day 46, and MLL-AF4 transcripts remained undetectable.E xtramedullary prophylaxis and re-induction with dexamethasone, cytarabine, etoposide, and asparaginase were completed; MRD stayed ≤0.005% and fusion transcripts stayed negative. Intensive and maintenance phases were finished, giving a total treatment duration of 146 weeks.1.5 years later, relapse was documented: bone marrow MRD was 0.11% and MLL-AF4 positivity was recorded. Blinatumomab and a relapse-protocol chemotherapy were given, but MRD and transcript levels continued to rise.Five years after diagnosis,CD19-CAR-T cells were infused, followed by CD22-CAR-T cells; complete molecular remission was restored. Allogeneic transplantation was declined by the family; maintenance was switched to oral chemotherapy. Treatment was discontinued, and the patient has remained molecularly negative and in good general condition through the last follow-up (18 October 2024).The treatment process for both individuals is shown in the Figure 4.

## Discussion

ALL is the commonest childhood leukaemia, with an incidence of 42 cases per million children (5) and an 80% cure rate for standard-risk phenotypes (6). In contrast to older-childhood acute lymphoblastic leukaemia, infant ALL still carries a dismal prognosis, and even cured patients frequently endure lifelong sequelae (7, 8). MLL-AF4, generated by t(4;11) (q21;q23), is the hallmark fusion gene of infant acute lymphoblastic leukaemia and operates as the principal oncogenic driver of the disease (9). Among monozygotic twins, the cumulative risk of concordant ALL reaches 10%—a rate orders of magnitude higher than in the general paediatric population. Since the nineteenth century, this striking predisposition has been attributed to monochorionic vascular anastomoses that create bidirectional “twin-to-twin” haematopoietic chimerism and allow pre-leukaemic clones to engraft in both siblings (10). Hematopoietic clones harbouring leukaemia-initiating mutations can cross from one twin to the other through placental anastomoses—a process known as the placental-transfer hypothesis. First proposed by Wolman (1962) (11) and later confirmed by molecular studies of concordant infant leukaemias, this mechanism explains the high rate of identical MLL-AF4 clones in monozygotic twins (12, 13). This report details the concurrent diagnosis and management of MLL-AF4 positive acute lymphoblastic leukaemia in monozygotic twins. The presence of a healthy 21-year-old sister, delivered 12 years earlier to the same parents, excludes vertical transmission of a fully penetrant leukaemogenic allele. Both infants had lived continuously since birth in a plastic greenhouse that emitted a pungent odour, indicating chronic exposure to volatile compounds released from polymer sheeting. Leveraging the genetic identity of monozygotic twins, their synchronous leukaemia provides a unique lens on pathogenesis. The shared MLL-AF4 fusion strongly implies that the initiating event occurred *in utero* or shortly after birth, establishing a pre-leukaemic ground state (14). Prolonged exposure to the malodorous atmosphere of the plastic greenhouse may then have functioned as a critical environmental trigger, damaging haematopoietic stem cells, perturbing gene expression and/or facilitating the t(4;11) translocation, thereby precipitating overt MLL-AF4 positive acute lymphoblastic leukaemia (15). Infant MLL-AF4 positive acute lymphoblastic leukaemia is highly aggressive and carries a poor prognosis because the fusion gene itself functions as a powerful leukaemogenic driver (16). Although intensive chemotherapy regimens—including the TOTXV regimen employed here—achieve high initial remission rates, molecular relapse remains frequent and long-term survival with conventional therapy is poor (17). Both twins followed a typical remission–relapse trajectory, underscoring the inadequacy of chemotherapy alone for this subset of patients.

Allogeneic haematopoietic stem-cell transplantation is an established modality for this disease. In China, however, its total cost—including post-graft immunosuppression—has been estimated at 200 000 CNY. CAR-T therapy is priced at approximately 100 000 CNY. Therefore, after economic evaluation, CAR-T treatment was selected by the parents. Over the past decade, chimeric antigen receptor T-cell (CAR-T) therapy has been developed through the genetic reprogramming of T cells to recognize tumor-associated antigens and thereby elicit a targeted immune response against malignant cells (18). This strategy provides a new therapeutic paradigm for hematologic malignancies. CD19 is an ideal CAR-T target in B-cell neoplasms because its expression is restricted to malignant B cells and their precursors, with negligible presence on essential non-B tissues (19). Grupp et al. (20) treated two pediatric patients with B-cell ALL using second-generation CD19 CAR-T cells. Approximately two months post-treatment, one patient experienced relapse with blast cells no longer expressing CD19, while the other patient achieved complete remission. In a separate CD19 CAR-T trial for CD19+ B-cell malignancies, 30 children with relapsed/refractory ALL attained complete remission (21). CD22 is under parallel clinical investigation and has emerged as an alternative target; CD22-directed CAR-T cells may confer therapeutic benefit to B-ALL patients ineligible for CD19-CART (22). Despite marked efficacy, single-target CAR-T is limited by antigen-negative relapse and acquired resistance. Dual CD19/CD22 CAR-T has therefore been explored. In a phase II study, 194 of 225 enrolled patients (<20 y) received concomitant CD19 and CD22 CAR-T cells, yielding a 99% complete response rate. Twelve-month overall survival was 87.7% and event-free survival (EFS) 73.5%. EFS was further improved in patients with B-cell aplasia or subsequent allogeneic stem-cell transplantation (SCT), indicating that dual targeting can achieve durable remission (23).

In this case, both paediatric patients with post-chemotherapy molecular relapse achieved deep and sustained molecular remission after sequential CD19 and CD22 CAR-T cell therapy followed by maintenance treatment. Despite not undergoing consolidation allogeneic hematopoietic stem cell transplantation due to economic reasons, both patients remained disease-free at the last follow-up (both aged 8 years). From a therapeutic mechanism perspective, dual-targeted CD19/CD22 CAR-T cell therapy precisely identifies and eliminates leukemia cells expressing corresponding antigens, effectively clearing residual tumor cells post-chemotherapy. This approach demonstrates targeted therapeutic advantages, particularly against drug-resistant or recurrent clones driven by the MLL-AF4 fusion gene (24). Subsequent maintenance therapy, involving the sustained use of low-intensity chemotherapy drugs, further suppresses the proliferation of potential residual leukemia cells, consolidates the efficacy of CAR-T therapy, and reduces the risk of disease recurrence (25). Neither child experienced secondary relapse after CAR-T and maintenance therapy, validating a “targeted clearance plus sustained suppression” paradigm. These observations indicate that, in selected MLL-AF4 infant ALL cases, deep molecular remission can be stabilised by maintenance alone, obviating the need for consolidative haematopoietic stem-cell transplantation (HSCT). This does not diminish the established value of HSCT; rather, it identifies an optimised maintenance regimen as a feasible alternative when HSCT is contraindicated, declined, or unnecessary because CAR-T has already achieved measurable residual disease–negative status. The inherent aggressiveness of MLL-AF4 infant ALL nonetheless mandates individualised risk stratification: in the presence of high-risk features such as extreme tumour burden or multidrug resistance, HSCT remains the most reliable curative option (26). Furthermore, the safety of long-term maintenance therapy was also validated in this case. Both pediatric patients experienced no serious adverse reactions during prolonged maintenance treatment, and their growth and development showed no significant impairment, indicating excellent tolerability of this maintenance regimen. These long-term outcomes challenge the conventional notion that transplantation is mandatory for such high-risk patients, suggesting that CAR-T cell therapy may offer a novel and effective long-term disease control pathway for patients who are ineligible or unsuitable for transplantation.

## Conclusion

This report delineates the clinical course of monozygotic twins with MLL-AF4 B-cell ALL who attained deep, sustained remission after chemotherapy followed by sequential CD19/CD22 CAR-T cell therapy, providing proof-of-concept that dual-target CAR-T can substitute for transplantation in selected patients. This case also illustrates the pronounced heterogeneity and high relapse risk of MLL-AF4 ALL, mandating long-term, prospective surveillance that couples minimal residual disease monitoring with comprehensive molecular profiling to enable precision therapy. Additional systematically documented cases are needed to refine risk-adapted strategies and further improve long-term outcomes for this rare leukaemia subset.

## Data Availability

The datasets presented in this study can be found in online repositories. The names of the repository/repositories and accession number(s) can be found in the article/Supplementary Material.
